# Optimising clinical governance and risk management in resource-limited hospitals: A family medicine model

**DOI:** 10.4102/phcfm.v17i1.4876

**Published:** 2025-04-08

**Authors:** Mergan Naidoo, Kimera T. Suthiram

**Affiliations:** 1 Department of Family Medicine, School of Nursing and Public Health, College of Health Sciences, University of KwaZulu-Natal, Durban, South Africa

**Keywords:** family medicine, clinical governance, risk management, resource-constrained healthcare, high-care unit, admissions protocol, patient safety, district hospitals

## Abstract

In resource-constrained healthcare settings, clinical governance and risk management are critical to improving patient outcomes and efficiently using limited resources. This article describes an innovative strategy implemented at a South African district hospital led by family physicians to optimise admissions and care prioritisation. The protocol established a designated high-care unit and admissions ward, ensuring that all new admissions were seen by a family physician, allowing family physicians to focus on the sickest patients requiring immediate intervention. This structured approach improved clinical oversight, reduced medical errors, and decreased morbidity and mortality. By efficiently allocating the expertise of family physicians, the intervention demonstrated measurable improvements in care delivery and patient safety. This model highlights the leadership role of family physicians in clinical governance and presents a scalable solution for similar resource-limited healthcare settings.

## Introduction

One of the global initiatives of the World Health Organization (WHO) is to provide Universal Health Care (UHC) through a primary health care (PHC) approach.^1^ Fundamental to the provision of UHC is the issues of quality, safety and governance using a cost-effective model. Clinical governance provides the framework for accountability in healthcare, ensuring that services are safe, effective and person-centred. Patient safety is central to UHC because unsafe care leads to avoidable harm, increased costs, and reduced trust in the health system. This approach enables inclusivity and provides patient care that is efficient and cost-effective. This international imperative requires the delivery of services by skilled healthcare workers to adopt the 2030 Sustainable Development Goals.^1^

Medical errors in South Africa’s low-resource PHC and district hospital settings are a significant concern, impacting patient safety and overall health outcomes. Several factors that contribute to these errors are discussed below:

### Resource constraints

Many health facilities face shortages of essential medical supplies and equipment, hindering the delivery of quality care. Inadequate infrastructure further exacerbates these challenges, leading to compromised patient safety.^2^

### Staffing shortages and training deficiencies

Understaffing and insufficient training are prevalent, resulting in overworked personnel who may lack the necessary skills to provide optimal care. This situation increases the likelihood of medical errors, including misdiagnoses and improper treatments.^3^

### Systemic challenges

Fragmentation of services and financial constraints impede the efficient delivery of healthcare. These systemic issues contribute to delays and errors in patient care, undermining the effectiveness of health services.^3,4^

### Impact on patient safety

The rise in medical errors has led to a surge in medicolegal claims, reflecting growing concerns about the quality of care. This trend underscores the need for comprehensive strategies to address the underlying causes of medical errors in these settings.^3,4^

These challenges are exacerbated by inadequate clinical governance structures, processes, activities and systems, leading to fragmented care, poor oversight, and increased morbidity and mortality. Contributory factors included the lack of experienced staff, the constant attrition of staff and reliance on medical interns for service delivery. Strengthening our health systems is crucial to ensure patients receive the highest attainable healthcare. Family physicians are uniquely positioned to address these gaps as generalists with leadership and systems-thinking skills.^5^ In addition, the current austerity measures introduced nationally within healthcare have had a crippling effect on patient care leading to innovative thinking to improve outcomes when resources are scarce.^6^

This article presents a family physician-led innovation implemented at a district hospital to demonstrate clinical governance and risk management in action. The intervention included the establishment of an admissions ward and a high-care unit to streamline patient care, optimise resource use, and prioritise the most critically ill patients. Historically, this intervention of implementing admissions and high-care units was not seen as a necessary addition to improving health outcomes. High-care units are not defined as entities in the District Hospital Package of Service and are considered resource-intensive. Most healthcare administrators have frowned at the idea of having high-care units at district hospitals and have been reluctant to support their creation. In recent years, the coronavirus disease 2019 (COVID-19) pandemic revealed the need for an intervention of this nature. The results of this initiative include reduced medical errors, decreased morbidity and mortality, and improved efficiency in clinical workflows.

## Context and problem statement

This 210-bed urban medium-sized district hospital, which serves a population of approximately 600 000, operates in a resource-limited environment. The hospital is staffed by medical interns, community service medical officers, medical officers and family physicians and offers the full package of service. It is characterised by the following:

- High volumes of patients with a mix of acute and chronic conditions. The hospital serves 600–800 outpatients a day through its ambulatory services.
- Large turnover of medical staff on a yearly basis.
- Limited family physicians and senior medical officers.
- Insufficient structures to prioritise and manage critically ill patients.
- A large number of patients who are admitted die within the first 48 h of admission.
- Increased medical errors and adverse outcomes because of fragmented admissions processes. Data on medical errors in the South African healthcare system is lacking, but experience with undergraduate and postgraduate student assignments has provided some insight into the frequency of such events.

Without structured clinical oversight, patients presenting with serious illnesses risk delays in care, improper triaging, and poor clinical outcomes.

## The intervention

**FIGURE 1:**
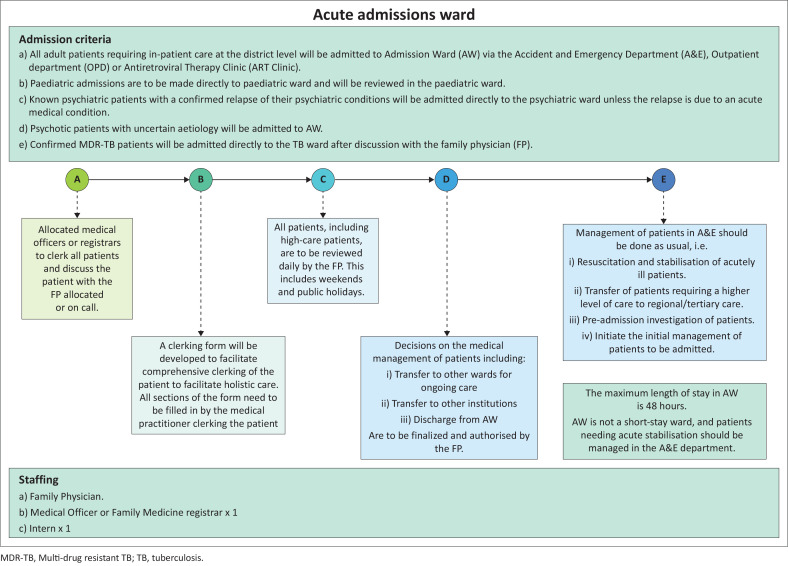

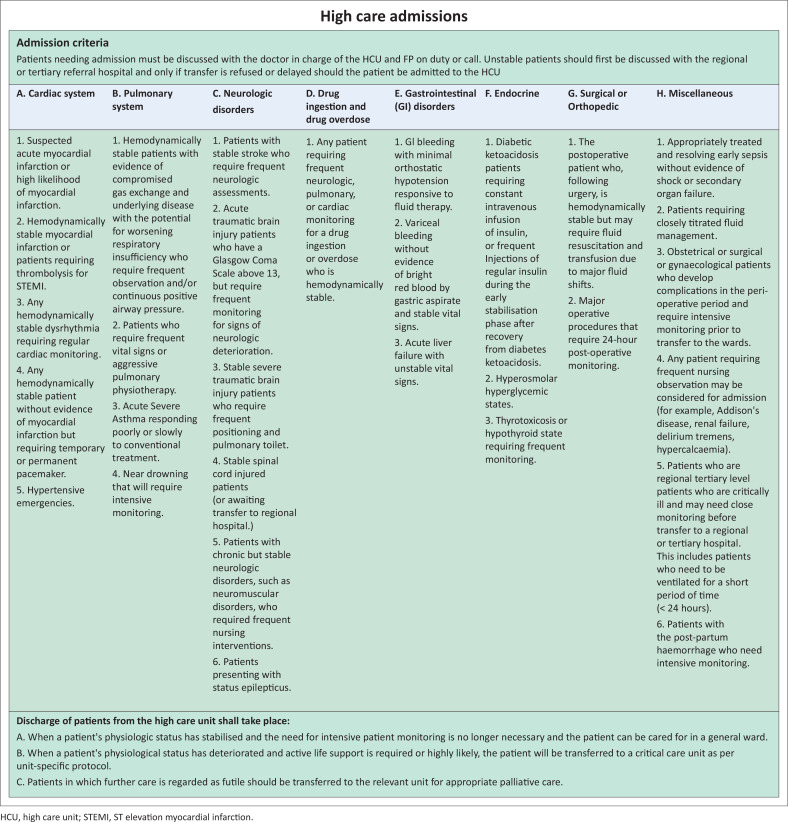
Guidelines designed by family physicians for the admissions ward and high care unit at a district hospital, KwaZulu-Natal, South Africa.

To address these challenges, a strategy for risk management was developed and implemented to better deal with the assessment, triage and management of patients. A high-care unit was created within the hospital for the sickest patients requiring close monitoring and urgent interventions, and an admissions ward where all new admissions were directed to a centralised ward. Family physicians prioritised critically ill patients, enabling focused, senior-level care. A family physician assessed and managed each patient during the critical stages of the admission process, that is, within the first 48 h. This ensured timely diagnosis, management planning, and appropriate triage to specialised units. The guidelines were designed for the admissions ward in a district hospital in KwaZulu-Natal (KZN), South Africa (Figure 1).

## Clinical oversight and resource allocation

The limited number of family physicians concentrated their efforts where their expertise was most needed: the sickest patients requiring admission and close monitoring. Once the patient was assessed, investigated, and a management plan was outlined, one of the four outcomes ensued:

- The discharge for an unwarranted admission.
- The development of a management plan and transfer to a general ward for ongoing care by junior colleagues.
- Discussion with specialists from regional or tertiary level of care and expedited transfer.
- Further stabilisation and admission to the high-care unit.

## Impact and outcomes

The protocol achieved significant improvements in patient care and clinical efficiency:

- **Reduced medical errors:** Family physicians picked up medical errors, such as a patient admitted with meningitis who was correctly diagnosed with postpartum eclampsia with an intracerebral bleed and was urgently transferred.
- **Efficient use of resources:** Family physicians could maximise their impact despite limited numbers by prioritising the sickest patients. Unwarranted admissions were discharged the next day, improving access to the limited number of beds and bed utilisation rates at the hospital.
- **Improved clinical workflows:** The structured admissions process reduced delays, improved triaging, and improved communication between healthcare teams.
- **Reduced morbidity and mortality:** Early identification and management of critically ill patients led to better outcomes in both morbidity and mortality and the crude mortality rates at the institution declined significantly.

Data collected from this hospital reflects one of the lowest crude death rates in the district in KZN. The crude death rate for 2018–2019 was reported to be 4.1%, in comparison to a crude death rate of 3.8% in 2019–2020.^7^ Quantitative data collected over the 12 months showed a measurable reduction in preventable adverse events, inpatient mortality, and length of stay. The experience of developing skills in managing acutely ill patients had a positive spin-off on the management of emergencies in the Accident and Emergency Department (A&E) and the High Care Unit (HCU). When confronted with the COVID-19 pandemic, this district hospital was able to quickly pivot on its inherent strengths and implement advanced respiratory support systems for very ill patients.^8,9^

## The role of family physician in advocating for effective clinical governance

Family physicians played a critical leadership role in:

- Designing and implementing a new model of care based on their lived experience at the hospital.
- Providing senior oversight to ensure quality care.
- Building capacity among junior staff and healthcare workers.
- Leading risk management efforts to reduce errors and adverse events and improve hospital efficiency indicators.

This highlights the unique position of family physicians to drive clinical governance initiatives in district hospitals, where specialist resources are limited.

## Lessons learned

Effective clinical governance systems can significantly improve patient safety and outcomes, even in resource-limited settings. As generalists with leadership skills, family physicians are well-suited to implement and oversee such systems. Prioritising admissions and critical care creates efficiency and reduces avoidable morbidity and mortality. Collaboration with junior doctors and other healthcare workers ensures sustainable implementation.

## Conclusion

Implementing a structured admissions ward and high-care unit demonstrates the value of family physicians in clinical governance and risk management. This model has the potential to be scaled up to other resource-constrained busy district hospitals, providing a pragmatic solution to improve patient outcomes and reduce medical errors. The role of family physicians in the South African public healthcare system remains unclear in many provinces, although they have made great strides in improving healthcare.^10,11,12^ Policymakers and hospital administrators should recognise and support the leadership role of family physicians in strengthening healthcare systems and ensuring quality, safe care for all patients.

## Call to action

The call to action includes:

- **For policymakers:** Invest in family physicians and clinical governance systems to improve hospital care and prevent an additional burden on regional and tertiary services. Consider replacing clinical managers at district hospitals with family physicians who have expertise in leadership and governance, clinical care, community-oriented primary care, and building capacity among the healthcare teams.^5^
- **For hospital administrators:** Adopt similar protocols to streamline admissions, triaging, and risk management.
- **For educators:** Incorporate clinical governance and leadership training into medicine curricula to prepare future leaders for resource-limited settings.
